# Maximizing temperature sensitivity in a one-dimensional photonic crystal thermal sensor

**DOI:** 10.1038/s41598-024-82889-4

**Published:** 2025-02-03

**Authors:** Manal A. Maher, Arafa H. Aly, Mohamed S. Esmail, S. E.-S. Abd El-Ghany

**Affiliations:** 1 Computed Tomography X-Ray Scan Unit, Cairo Egyptian Museum, Ministry of Antiquities, Cairo, Egypt; 2 TH‑PPM Group, Physics Department, Faculty of Sciences, Beni-Suef University, Beni Suef, 62514 Egypt; 3 Basic Science Department, Faculty of Engineering, Misr University for Science and Technology (MUST), Giza, Egypt; 4 Physics Department, Faculty of Sciences, Benha University, Benha, Egypt

**Keywords:** 1D Photonic crystal, Thermal sensor, Photonic bandgap, Thermo-optic effect, Materials science, Mathematics and computing, Nanoscience and technology, Optics and photonics, Physics

## Abstract

This paper focuses on a defective one-dimensional photonic crystal thermal sensor with fabricated layers of gallium nitride, glycerin, and air. The transmission features of this sensor have been presented based on the transfer matrix approach using MATLAB software. Interest in the sensor’s sensitivity to temperature variation is for the sake of the photonic bandgap behavior of the 1D photonic crystal and the thermo-optic effect of glycerin must be preserved over a long time in protecting archaeological artifacts. In this direction, theoretical modeling together with numerical simulation studies are conducted to optimize the refractive index of GaN to enhance sensitivity. This work is going to evaluate the performance of the sensor in terms of the shift in the transmission spectrum of the sensor with the imposition of changes in temperature. The effect of the thickness of the defect layer together with the incident angle on the performance of the sensor will be discussed further. Sensor sensitivities are about 10 nm/°C, with a quality factor reaching a high value of 35,443 at an incident angle of 30°, while sensitivities at an incident angle of 65° have 20 nm/°C and a quality factor of 14,723.

## Introduction

Photonic crystals (PhCs) are a novel optical material with significant research potential due to their unique applications, which can be enhanced by controlling their structure^1–4^. A PhC is an optical nanostructure with periodic changes in refractive index, affecting light propagation, similar to how natural crystals create electronic band structures^5–7^. Photonic band gaps (PBGs) are structural coloration animal reflectors found in nature. Since Eli Yablonovitch^7^ and Sajeev John’s^5^ 1987 work, research on PhCs has grown, with a focus on their sensing applications, offering unique capabilities for high-sensitivity devices. PhCs can be used as excellent optical sensors such as biochemical sensors^8–11^, mechanical sensors^12^, temperature sensors^13–15^, chemical sensor^16^, refractive index (RI) sensors^17,18^, and gas sensors^19,20^. They are also used in the applications of light flow control, such as optical fibers,

waveguides, and optical filters^21–24^. Additionally, PhCs could be used for angular velocity sensing^25^. PhCs, fabricated in various dimensions, are optical materials used for controlling light flow and manipulating it. They exhibit photonic band gaps, limiting light propagation, and are extensively researched for high-sensitivity sensing devices^26^.

One-dimensional photonic crystals (1D PhCs) modulate their refractive index by alternating layers of materials with different indices. They have applications in silicon photonics, self-collimation, negative refraction, optical diodes, light bending, and sensing devices doped with bio-active metals like silver, which are proposed as sensing devices for bacterial contaminants^27^. The technology has potential applications in high-efficiency solar cells and optoelectronic devices, including angle sensors for optical fiber systems and polarization-sensitive couplers^28^. The subject has been extensively researched in thermal sensors, sensing platforms, photovoltaic devices, and full color displays^29–32^. The temperature sensors’ sensing principle involves the change in photonic band gap due to the variation in refractive index with temperature^29^. The applications showcase the versatility and potential of 1D PhCs in various fields such as photonics, sensing, and solar energy. Temperature sensors utilize photonic band gap changes due to refractive index variation, demonstrating the versatility and potential of 1D PhCs in photonics, sensing, and solar energy.

Researchers are increasingly interested in the 1D photonic crystal thermal sensor due to its potential applications and varying sensing parameter values. In 2012, Kumar et al.^33^ proposed a simple temperature sensor design using a 1D PhC with a silicon defect mode, consisting of a Si/air multilayers system, displaying a linear shift in sensitivity of 0.064 nm/K across a 100–700 K temperature range. In 2012, Chang et al. studied the temperature dependence of defect mode in a defective one-dimensional photonic crystal, focusing on the visible region. They designed a 1D PhC with TiO_2_ and SiO_2_ as defects, enhancing sensitivity by 35% at 100 °C. This design can be used as a tunable filtering device and a temperature-dependent optical sensing device. Chang et al.^13^ conducted a study in 2012 on the temperature dependence of defect modes in a defective one-dimensional photonic crystal. They designed a 1D PhC using TiO_2_ and SiO_2_, with SiO_2_ and Bi_4_Ge_3_O_12_ as defect layers. The design improved sensitivities of 0.37 nm and 0.50 nm at a temperature increase of 100 °C by 35%, making it suitable for tunable filtering and temperature-dependent optical sensing. Zhang^34^ developed a high-sensitivity temperature sensor in 2016 using PhC nanobeam cavities, achieving a sensitivity of 162.9 pm/k and a quality factor of 4 × 10^4^, making it suitable for small-diameter on-chip systems. Elmahdy et al.'s^15^ 2018 design for a 1D PhC thermal sensor increased sensitivity with temperature and defect thickness, reaching 0.328 nm/°C for TE mode and quality factor value 4840. In 2018, El-Amassi et al.^35^ proposed a 1D PhC with a polymer layer between Si and SiO_2_, enhancing temperature sensor sensitivity of 0.380 nm per degree rise in temperature. The study found that polymers like polycarbonate and polystyrene significantly improved the temperature-sensitive transmission peak shift, emphasizing the importance of polymer choice in temperature sensing. Abd El-Ghany^14^ presented an interest paper discusses the use of 1D PhCs with double defects as temperature sensors, highlighting their potential for practical temperature sensing applications. Chen et al.'s^36^ 2019 PhC structure successfully achieved 88.7 pm/°C temperature sensitivity and 471.4 quality factor value for simultaneous temperature and gas concentration sensing. El-Naggar et al.'s^37^ 2020 study on temperature dependency of defect modes in cylindrical photonic crystals (CPhCs) found that the structure, composed of TiO_2_ and Al_2_O_3_ shells with a single defect layer, showed tunability and peak transmittance sensitivity of 0.0056 nm/ºC. In 2020, Abadla et al.^38^ conducted a study on the thermal properties of a one-dimensional annular PhC with nematic liquid crystal as a defect circular layer. The study used TMM to analyze transmission spectrum and optimize parameters, revealing a sensitivity of 0.167 nm/°C and 0.224 nm/°C for two defect modes. Abadla et al.'s^39^ 2021 study utilized the transfer matrix method to investigate the thermal properties of one-dimensional defective annular photonic crystals, resulting in a temperature sensor with a sensitivity of 0.01069 nm/°C. Elsayed et al.^40^ developed a temperature sensor using a one-dimensional photonic crystal with graphene monolayer defect layer on nematic liquid crystal. The sensor’s sensitivity increased with thicker defect layer, reaching 4 nm/°C and quality factor up to 11,000 at 2500 nm thickness. Zaky and Arafa^41^ proposed a temperature and salinity sensor in 2021 using a 1D-PhC platform and Tamm resonance. The sensor has high sensitivity (from 2.8 to 10.8 nm/°C), a high-quality factor, and a low detection limit of 3 × 10^−7^ nm. Thara et al.^42^ proposed temperature sensors in 2022 using 1D random photonic crystals at different temperatures and binary and ternary-based multi-layered structures. The sensor configurations included (Si/SiO_2_)^N^, (Si/SiO_2_/TiO_2_)^N^, and (Si/SiO_2_/PS)^N^ at N = 10. The 1D PhC structure with a ternary-based structure shows maximum wavelength shifts and maximum sensitivity of 0.08758 nm/°C when using Polystyrene instead of TiO_2_. Kumar and Prasad^43^ developed a sensor in 2022 that combines graphene and hexagonal boron nitride for improved sensing performance. The sensor can detect temperature changes in various ranges, with 10^10^ detection accuracy at room temperature and 372,176 high-quality at 49° angle. The device has a 5K detection limit and 0.20pm/K sensitivity with 50-unit cells, which can be expanded to 300K to 900K. Aly et al.^30^ presented a temperature sensor in 2023 using a one-dimensional photonic crystal structure with alternating silicon and porous silica layers. The sensor effectively detects temperature changes, with a quality factor of 2216.6 and sensitivity of 0.085 nm/°C. Also in 2023, Srivastava^44^ studied the improvement of temperature sensitivity using a one-dimensional (1D) symmetric defective photonic crystal (PC) made of Si and Bi_4_Ge_3_O_12_ material. The study found that the temperature sensitivity increased by 0.173 nm/°C for symmetric defective PC with an external defect layer and 0.140 nm/°C for symmetric defective PC without an external defect layer. The highest sensitivity (0.173 nm/°C) was achieved with the symmetric defective PC containing an external defect layer. The study also found that symmetric defective 1DPCs with defects have a higher Q factor, with the highest quality factor reaching 2284 at 375 °C. The proposed design is affordable, simple to create, and small in dimension. Recently, in 2024 Bouazzi et al.^45^ introduces a new 1D topological photonic crystal (PC) mirror heterostructure for advanced thermal sensing. It utilizes a coupled topological edge state mode (CTES) with exceptional light confinement and a high quality (Q) factor. The first NLC configuration achieves an outstanding Q factor of 10^7^, surpassing the intrinsic value. Additionally, it demonstrates exceptional thermal sensitivity (− 0.12317 nm/°C) and a remarkable figure of merit (1002.48 °C^−1^). The second configuration offers a trade-off between sensitivity and tunability, exhibiting a Q factor in the 10^6^ range, a sensitivity of − 0.05853 nm/°C, and a figure of merit of 86.53 °C^−1^. This platform holds immense promise for developing high-performance, tunable narrowband filters and ultrasensitive thermal sensors, paving the way for advancements in diverse photonic applications.

## Design consideration

### Structure without defect

**Fig. 1:**
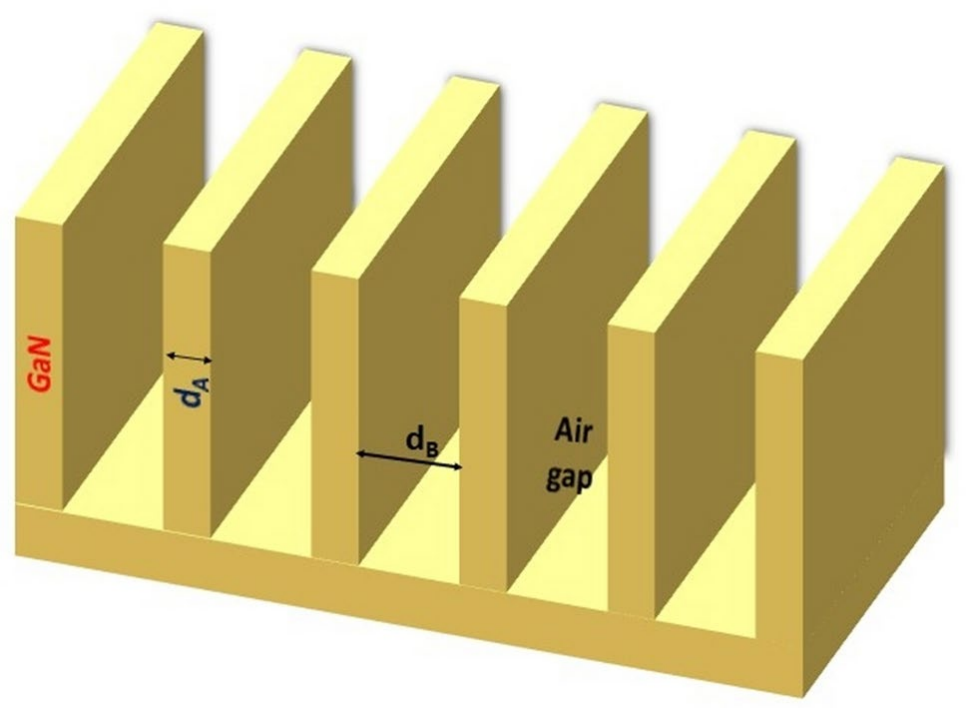
Shows a schematic representation of the 1D PC structure of GaN-air without glycerin defect.

Figure 1 shows a schematic representation of the proposed finite size 1D PhC of structure periodic cells (GaN/air) without glycerin defect material. The periodic cells consist of Gallium nitrite and air with thicknesses of d_A_ = 100 nm and d_B_ = 200 nm respectively. GaN and air have been taken as the high and the low refractive indices (RI) media. The RI of GaN is 2.27756^46^ and of air is 1.

**Fig. 2:**
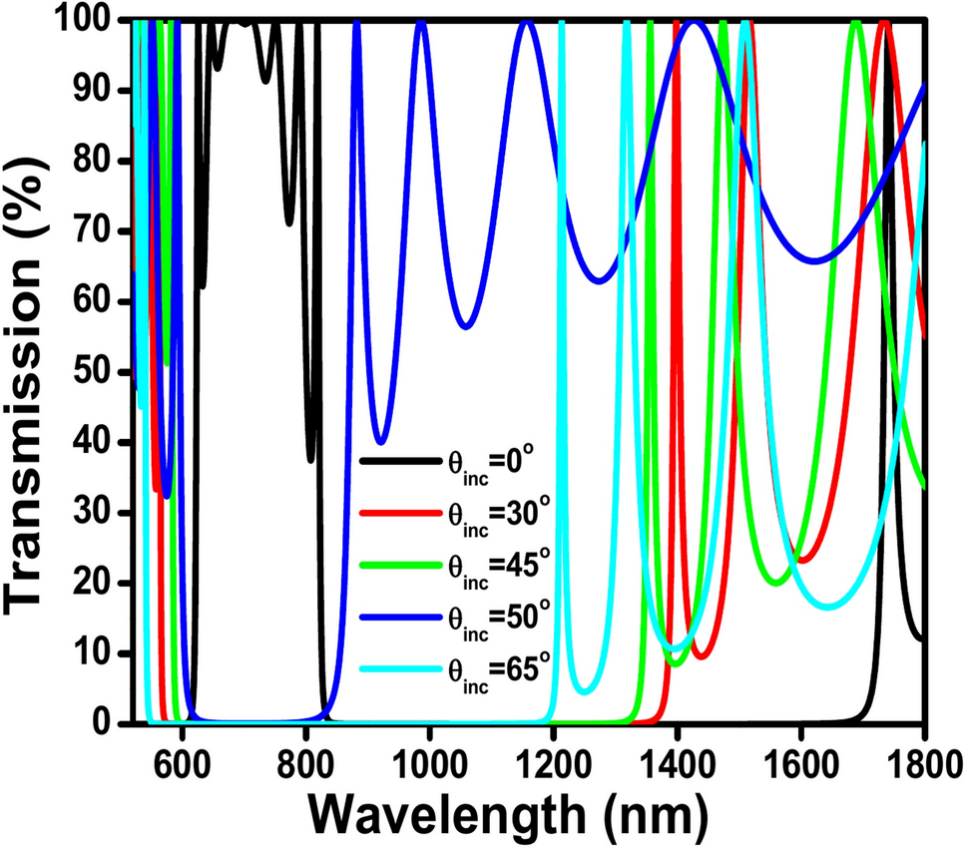
Shows the transmission spectra of the non-defected PC (GaN/air) for TE mode at incident angles 0°, 30°, 45°, 50°, 65° respectively.

Gallium Nitride (GaN) is a dielectric material that has been studied for its potential applications in PhC sensors^47^. It exhibits high electron mobility, wide energy band gap, and excellent thermal properties, making it suitable for various sensing applications, including as a temperature sensor in PhC sensors. It also has excellent thermal properties, making it suitable for various sensing applications, including as a thermal sensor. GaN’s high thermal conductivity, which is about 1.3 W/cmK, allows for efficient heat dissipation, enabling accurate temperature measurements^48^. Additionally, GaN’s stability at high temperatures, biocompatibility, and chemical stability make it a highly reliable material for thermal sensing^47^. These properties have led to the development of GaN-based temperature sensors, such as p-GaN/AlGaN/GaN hybrid anode diodes, which have demonstrated wide operation temperatures from 73 to 573 K, showcasing the potential of GaN in high-performance temperature sensing applications^49^. Also, GaN-based humidity sensors have been fabricated through pulse modulated DC magnetron sputtering, and GaN nanostructures have been used for gas sensing applications^47,50^. GaN is also used in the production of dielectric power devices, and GaN-based field effect transistor (FET) type gas sensors have been developed^51^, highlighting the versatility of GaN in sensing technologies, including thermal and gas sensing applications. Research has been conducted on the fabrication of GaN PhCs with photonic band gaps in the visible and infrared wavelength regimes for future optical characterization^52–54^. The use of GaN in PhC sensors demonstrates its potential for advanced sensing technologies with promising practical implications. The transmission spectra of the non-defected PhC (GaN/air) for TE mode at incident angles 0°, 30°, 45°, 50°, and 65° respectively are shown in Fig. 2.

### Structure with central defect

**Fig. 3:**
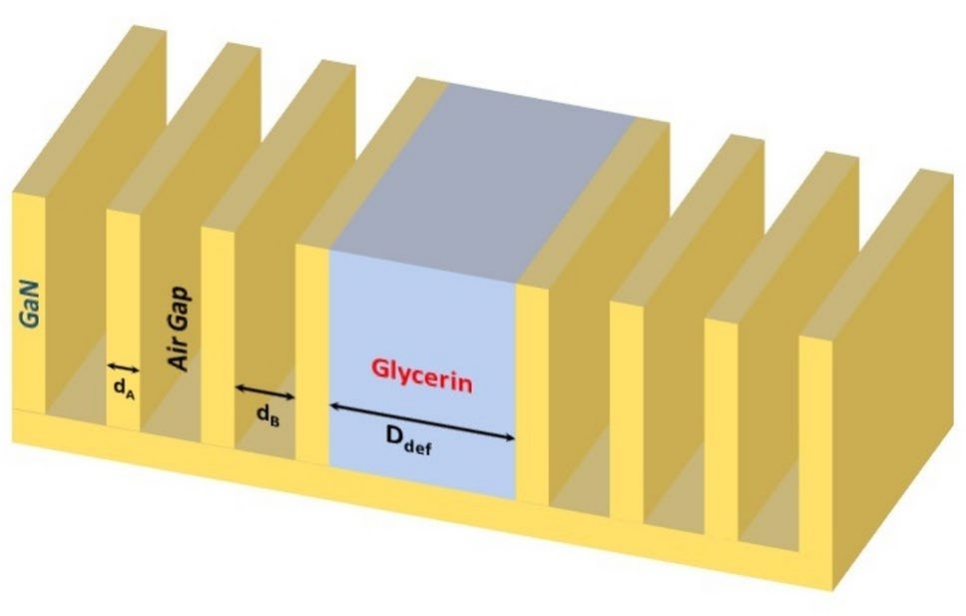
Shows a schematic representation of the 1D PC structure of [(GaN/Air)^3^GaN/Gylecrin/GaN(Air/GaN)^3^] with central defect of glycerin as temperature sensing material.

Figure 3 shows a schematic representation of the proposed finite size 1D PhC of structure periodic cells (GaN/air) with glycerin central defect material at periods repetitions N = 3 [(GaN-Air)^3^ (glycerin) (GaN-Air)^3^]. While D_def._ is the thickness of the glycerin defect layer. The glycerin was used as temperature sensing material because its reflective index decreases as temperature increases according to the equation^55^.

1 $${n}_{G}= {n}_{o}-\alpha \left(T-{T}_{o}\right)$$

where, ${n}_{G}$ is the refractive index at temperature T, n₀ is the refractive index at the reference temperature and α is the thermo-optic coefficient (how much the refractive index changes with temperature).

Glycerin is commonly used as a temperature-sensitive material in PhC temperature sensors. It is known for its low thermal expansion coefficient, making it suitable for temperature sensing applications. Research has demonstrated the use of glycerin in various sensor configurations, such as selectively infiltrated PhC fibers, where it has shown compatibility with temperature sensing and polarization filtering. Additionally, glycerol microdroplets have been utilized as high-sensitivity whispering gallery mode humidity sensors, showcasing their potential for temperature-independent optical sensing. The unique properties of glycerin, including its low thermal expansion coefficient, make it a promising material for temperature sensing in PhC sensors^56,57^.

## Theoretical considerations

### Transfer matrix method (TMM)

In this study, we used the transfer matrix method (TMM) to simulate the optical spectra of the photonic structures in question. TMM is a numerical method based on Abeles’s approach that can extract the transmission and reflection spectra of a multilayer structure. Yeh^58^ introduced the transfer matrix method (TMM) as an appropriate numerical method to analyze electromagnetic wave transfer in multilayered media and extract the transmission and reflection spectra of a multilayer structure. This method is suitable for resolving standard problems in the photonic band structure transmission spectrum. It is based on Abele’s approach^59^, which lets us show how the amplitudes of the electric fields of the incident wave ${\varepsilon }_{0}^{+}$, the reflected wave ${\varepsilon }_{0}^{-}$, and the transmitted wave change after m layers ${\varepsilon }_{m+1}^{+}$. The following matrix for layered films within m layers was created to achieve this:

2 $$\left(\begin{array}{c}{\varepsilon }_{o}^{+}\\ {\varepsilon }_{o}^{-}\end{array}\right)=\prod_{j=1}^{m+1}{C}_{j}\left(\begin{array}{c}{\varepsilon }_{m+1}^{+}\\ {\varepsilon }_{m+1}^{-}\end{array}\right)$$

*Cj* is the propagation matrix with the matrix elements:

3 $${C}_{j}=\left[\begin{array}{cc}\frac{1}{{\tau }_{j}}{e}^{i{\phi }_{j-1}}& \frac{{\rho }_{j}}{{\tau }_{j}}{e}^{-i{\phi }_{j-1}}\\ \frac{{\rho }_{j}}{{\tau }_{j}}{e}^{i{\phi }_{j-1}}& \frac{1}{{\tau }_{j}}{e}^{-i{\phi }_{j-1}}\end{array}\right]$$

where ${\phi }_{j-1}$ indicate the change in the phase of the wave between the (*j*-1)th and the *j*th interfaces and are expressed in the following equations:

4 $$\left\{\begin{array}{l}{\phi }_{0}=0 \text{for} j=1\\ {\phi }_{j-1}=\frac{2\pi }{\lambda } {\widehat{n}}_{j-1}{d}_{j-1}\text{cos}{\Theta }_{j-1}\text{ for } j>1\end{array}\right.$$

$\lambda$ is the wavelength for the incident light in vacuum and ${d}_{j-1}$ is the thickness of the (*j*-1)th layer.

The values $\tau$_j_ and $\rho$_j_ are the Fresnel transmission and reflection coefficients, respectively, between the (*j*-1)th and the *j*th layers. These coefficients can be expressed using the complex refractive index $\widehat{n}= {n}_{j}+{ik}_{j}$ and the complex refraction angle ${\Theta }_{j}$.

For TE polarization:

5 $${\rho }_{j}^{TE}= \frac{{\widehat{n}}_{j-1}\text{cos}{\Theta }_{j}- {\widehat{n}}_{j}\text{ cos}{\Theta }_{j-1} }{{\widehat{n}}_{j-1}\text{cos}{\Theta }_{j}+ {\widehat{n}}_{j}\text{ cos}{\Theta }_{j-1} }$$

6 $${\tau }_{j}^{TE}= \frac{{2\widehat{n}}_{j-1}\text{cos}{\Theta }_{j-1} }{{\widehat{n}}_{j-1}\text{cos}{\Theta }_{j}+ {\widehat{n}}_{j}\text{ cos}{\Theta }_{j-1} }$$

Besides, for the TM polarization:

7 $${\rho }_{j}^{TM}= \frac{{\widehat{n}}_{j-1}\text{cos}{\Theta }_{j-1}- {\widehat{n}}_{j}\text{ cos}{\Theta }_{j} }{{\widehat{n}}_{j-1}\text{cos}{\Theta }_{j-1}+ {\widehat{n}}_{j}\text{ cos}{\Theta }_{j} }$$

8 $${\tau }_{j}^{TM}= \frac{{2\widehat{n}}_{j-1}\text{cos}{\Theta }_{j-1} }{{\widehat{n}}_{j-1}\text{cos}{\Theta }_{j-1}+ {\widehat{n}}_{j}\text{ cos}{\Theta }_{j} }$$

The complex refractive indices and the complex angles of incidence obey Snell’s law, for j = 1,2, 3, . . . m + 1:

9 $${\widehat{n}}_{j-1}\text{sin}{\Theta }_{j-1}= {\widehat{n}}_{j}\text{ sin}{\Theta }_{j}$$

Since there is no reflection from the final phase, ${\varepsilon }_{m+1}^{-}$. Abele’s obtained then a conventional formula for the total reflection and transmission coefficients corresponding to the amplitude reflectance ρ and transmittance τ, respectively, as follows:

10 $$\rho =\frac{{\varepsilon }_{0}^{-}}{{\varepsilon }_{0}^{+}}=\frac{{c}_{21}}{{c}_{11}}$$

11 $$\tau =\frac{{\varepsilon }_{m+1}^{+}}{{\varepsilon }_{0}^{+}}=\frac{{\tau }_{1}{\tau }_{2}{\tau }_{3}\dots \dots ..{\tau }_{m+1}}{{c}_{11}}$$

The quantities ${c}_{11}$ and ${c}_{21}$ are the matrix elements of the whole product ${c}_{j}$ matrix:

12 $${C}_{1} {C}_{2}{C}_{3}\dots \dots {C}_{m+1}=\left(\begin{array}{cc}{c}_{11}& {c}_{12}\\ {c}_{21}& {c}_{22}\end{array}\right)$$

For TM and TE polarizations and the transmission T is:

13 $${T}_{TM}=Real \left(\frac{{\widehat{n}}_{m+1}\text{cos}{\Theta }_{m+1} }{{\widehat{n}}_{0}\text{cos}{\Theta }_{0} }\right) {\left|{\tau }_{TM}\right|}^{2}$$

14 $${T}_{TE}=Real \left(\frac{{\widehat{n}}_{m+1}\text{cos}{\Theta }_{m+1} }{{\widehat{n}}_{0}\text{cos}{\Theta }_{0} }\right) {\left|{\tau }_{TE}\right|}^{2}$$

While real indicates the real part.

### Sensor’s characterizations

The sensitivity (S), quality factor (QF), figure of merits (FOM), signal-to-noise ratio (SNR), detection limit (δn) and sensor resolution (SR) are also important sensing parameters to evaluate sensor performance. These parameters can be found using the following expressions: The sensitivity (S) is defined as the ratio of the change in resonant wavelength ($\Delta {\lambda }_{res})$ to the change in temperature (Δ*T*), it could be written as $\left(S= \frac{{\Delta \lambda }_{res}}{\Delta \text{T}}\right)$, and it is measured in the unit of *nm*/℃. The quality factor (QF) of the suggested thermal sensor is calculated using the resonance wavelength ${\lambda }_{res}$ and the full width at the half maximum of the output transmission peak Δλ_FWHM_ as given by $\left(QF= \frac{{\lambda }_{res}}{{\Delta \lambda }_{FWHM}}\right)$^15^. The figure of merit (FOM) is often calculated as the ratio of the sensitivity (S) to the bandwidth Δλ_FWHM_ of the resonant peak^30^ and given by $\left(\text{FOM}= \frac{S}{{\lambda }_{FWHM}}\right)$. FOM is used for the evaluation on sensing performance of RI sensors^60^. Moreover, FOM provides a measure of the sensor’s performance in detecting temperature variations and it is measured by inverse of the degrees Celsius (°C^−1^) or Kelvin (K^−1^). The signal-to-noise ratio (SNR) is defined as absolute of the change in resonant wavelength (Δλ_*res*_) to change of the full width at the half maximum of the output transmission $\Delta {\lambda }_{FWHM}$ as given by $\left(\text{SNR}=\left|\frac{{\Delta \lambda }_{res}}{{\Delta \lambda }_{FWHM}}\right|\right)$. The detection limit (δn) of a one-dimensional photonic crystal (1D PhC) thermal sensor refers to the smallest detectable change in a temperature that the sensor can reliably measure^30^. The detection limit is determined by its sensitivity and the smallest temperature change it can accurately measure and given by $\left(\updelta \text{n}= \frac{1}{S} \frac{{\Delta \lambda }_{FWHM}}{1.5 \left({SNR}^{0.25}\right)}\right)$ and it is measured by a temperature unit (ºC)^61^. Additionally, the detection limit (δn) is a crucial parameter that indicates the precision of the sensor in detecting the temperature variations. The measuring unit for the detection limit is typically degrees Celsius (°C) or Kelvin (K). The sensor resolution (SR) is defined as the minimal variation of the refractive index (RI) that can be detected by the sensor and it is given by multiply of the detection limit (δn) and the sensitivity (SR= $\left|\updelta {\text{n}} \cdot \text{S})\right|$^62^.

## Results and discussions

### Effect of change glycerin defect thickness (D_def_)

**Fig. 4:**
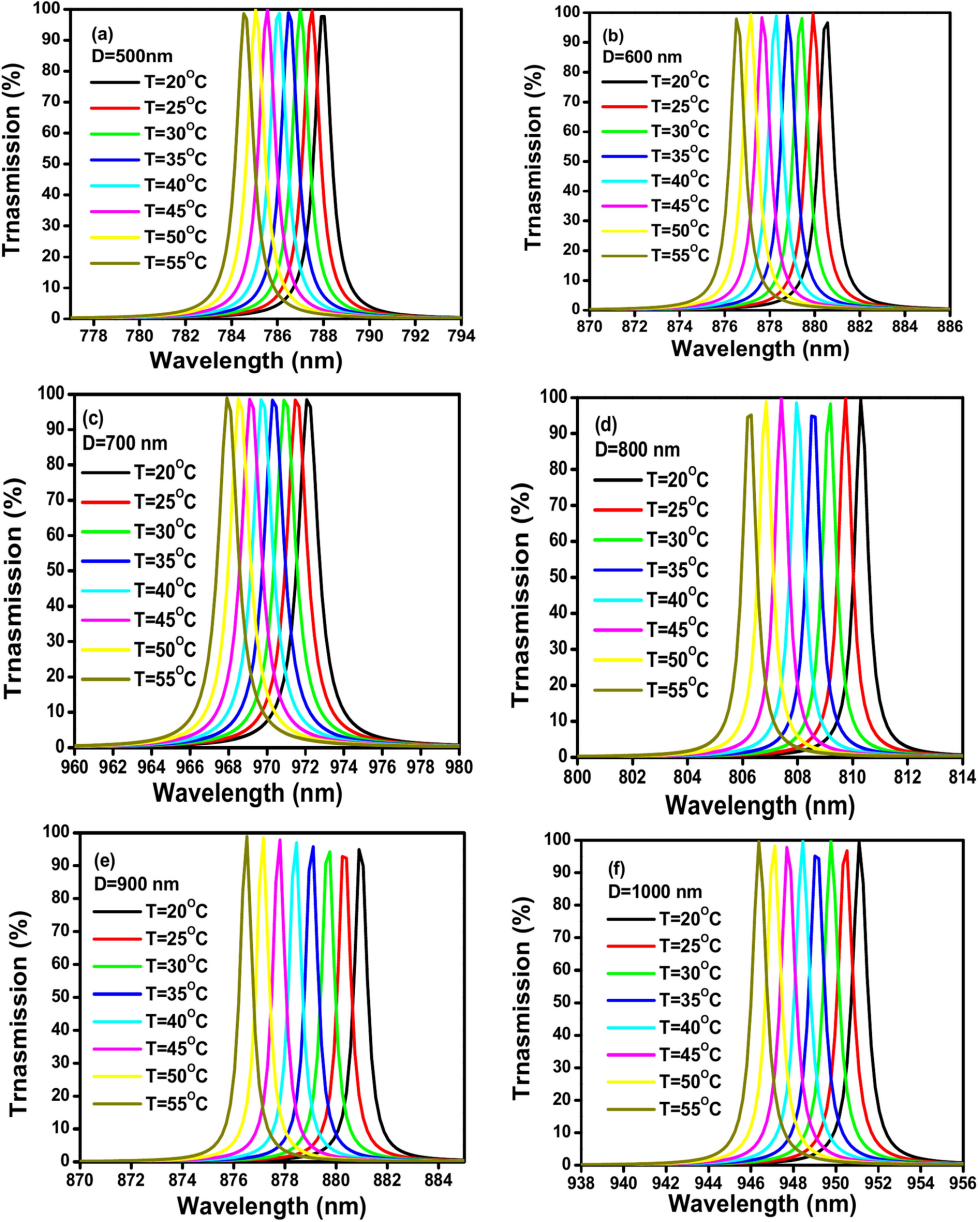
(**a**–**f**) Shows the TE transmission spectra of the proposed 1D PC thermal sensor (GaN/air) with defect thicknesss D_def_ from 500 to 1000 nm with a step of 100 nm. With parameters: an incident angle θ = 0º, GaN thickness d_A_ = 100 nm, air thickness d_B_ = 200 nm and number of GaN/air periods N = 3: (**a**) D = 500 nm, (**b**) D = 600 nm, (**c**) D = 700 nm, (**d**) D = 800 nm, (**e**) D = 900 nm, and (**f**) D = 1000 nm.

Figure 4a–f display TE transmission spectra of the suggested 1D PhC (GaN/air) thermal sensor at glycerin different defect thicknesses D_def_ from 500 to 1000 nm with a step of 100 nm. With parameters as follows: an incident angle θ = 0º, GaN thickness d_A_ = 100 nm, air thickness d_B_ = 200 nm and number of GaN/air period repetitions N = 3. The figures show an overlap between the peaks at various temperatures which indicates to reduce the sensitivity. In addition, the broad peaks at different temperatures from 20 to 55 ºC indicate a reduction of the quality factor.

Table 1 shows the values of different sensing parameters of the proposed thermal sensor that were calculated at different central glycerin defect thicknesses. The sensitivity (S), quality factor (QF), figure of merit (FOM), signal-to-noise ratio (SNR), detection limit (δn), and sensor resolution (SR) are calculated of each thickness D_def_ from 500 to 1000 nm with a step of 100 nm. The temperature range, wavelength range and shift direction also are identified for each thickness. According to Table 1's data, it seems that the values of sensitivity (S) and figure of merit (FOM) that follow an increase in defect thickness (D_def_) are positively correlated. As the thickness of the defect increases from 500 to 1000 nm, the sensitivity increases from 0.099 to 0.135 nm/°C. Also, FOM value rises from 0.116 to 0.219 °C^−1^ when the defect thickness increases from 500 to 900 nm. At 1000 nm defect thickness, FOM slightly decreases (0.169 °C^−1^). Although this slight decrease of the FOM’s value at D_def_ 1000, it remains higher than the FOM values at lower thicknesses (D_def_) from 500 to 700 nm. Noteworthy, the quality factor (QF) of the proposed 1D PhC thermal sensor doesn’t follow a clear trend when increasing the glycerin defect thickness. Whereas it increases significantly D_def_ from 500 to 600 nm and then decreases from D_def_ 600 nm to 700 nm, which is the lowest value of 795.662 at D_def_ 700 nm. After that, it increases again from D_def_ 700 nm to 900 nm, which reaches the highest value of the QF of the proposed sensor 1539.678. At last, the quality factor drops a little between (D_def_) 900 and 1000 nm. Furthermore, there is no discernible correlation between the signal-to-noise ratio (SNR) and the defect thickness D_def_. Whereas SNR doesn’t consistently increase or decrease with increasing the glycerin defect thickness. It fluctuates significantly across the different thicknesses. It increases from D_def_ 500 nm to 600 nm, then it decreases dramatically at D_def_ 700, which represented the lowest value of SNR of 95.955 comparing with the other SNR’s values in the table. Then, it significantly increased from D_def_ 700 nm to 800 nm, which reaches the highest value of SNR at D_def_ = 800 nm of 671.001. Finally, SNR significantly decreased from D_def_ = 800 nm to 1000 nm. Also, there appears a complex relationship between the increase in the defect thickness D_def_ and the detection limit (δn) of the proposed 1D PhC thermal sensor. It fluctuates significantly across the different thicknesses by increasing and decreasing. It decreases from D_def_ 500 nm to 600 nm, then it increases significantly at D_def_ 700, which is the highest value of δn 1742.350 ºC. After that, it significantly decreased from D_def_ 700 nm to 800 nm, to reach the lowest δn’s values in the table 906.304 ºC. Finally, δn significantly increased from D_def_ 800 nm to 1000 nm. With respect to the relation of the glycerin defect thickness and the sensor resolution (SR) of the proposed sensor. It is noticed, SR’s values slightly increased from D_def_ 500 nm to 600 nm, then it increases significantly at D_def_ 700, which is the highest value of SR 206.555 nm compared with the other SR’s values in the table. After that, it decreased from D_def_ 700 nm to 800 nm, to reach the lowest SR’s values in the table 107.442 nm. Finally, SR significantly increased from D_def_ 800 nm to 1000 nm to reach the value 155.975 nm which is also higher than the SR values at lower thicknesses D_def_ from 500 to 600 nm. All the TE transmission spectra occurred at a temperature range from 20 to 55 °C. Furthermore, all the resonances have a blue shift direction in the beginning of the near-infrared region from 780 to 980 nm. Thus, based on the interpretation of the effect of the change in the defect thickness D_def_ in the sensing parameters of the suggested thermal sensor, D_def_ 900 nm is an optimal glycerin defect thickness for maximizing the sensitivity and quality factor of this specific sensor design.

**Table 1:** Shows the values of the sensitivity (S), quality factor (QF), figure of merit (FOM), signal-to-noise ratio (SNR), detection limit (δn), and sensor resolution (SR) by increasing the glycerin central defect thickness D_def_ from 500 to 1000 nm, at an incident angle θ = 0º, GaN thickness d_A_ = 100 nm, air thickness d_B_ = 200 nm and number GaN/air of periods N = 3 with identification the temperature range, wavelength range and shift direction of each thickness.

D_def_	S (nm/ºC)	QF	FOM (ºC^−1^)	SNR	δn (ºC)	SR (nm)	Temp. range	Wavelength range (nm)	Shift direction
500	0.098	922.314	0.116	234.037	1356.675	134.215	20–55	780–792	Blue
600	0.114	1169.140	0.152	364.870	1175.792	134.310	20–55	872–886	Blue
700	0.118	795.662	0.097	95.955	1742.350	206.555	20–55	960–980	Blue
800	0.116	1387.554	0.201	671.001	906.304	107.442	20–55	802–814	Blue
900	0.125	1539.678	0.219	453.342	1016.438	120.498	20–55	873–884	Blue
1000	0.135	1184.781	0.169	160.395	1315.690	155.975	20–55	942–956	Blue

**Fig. 5:**
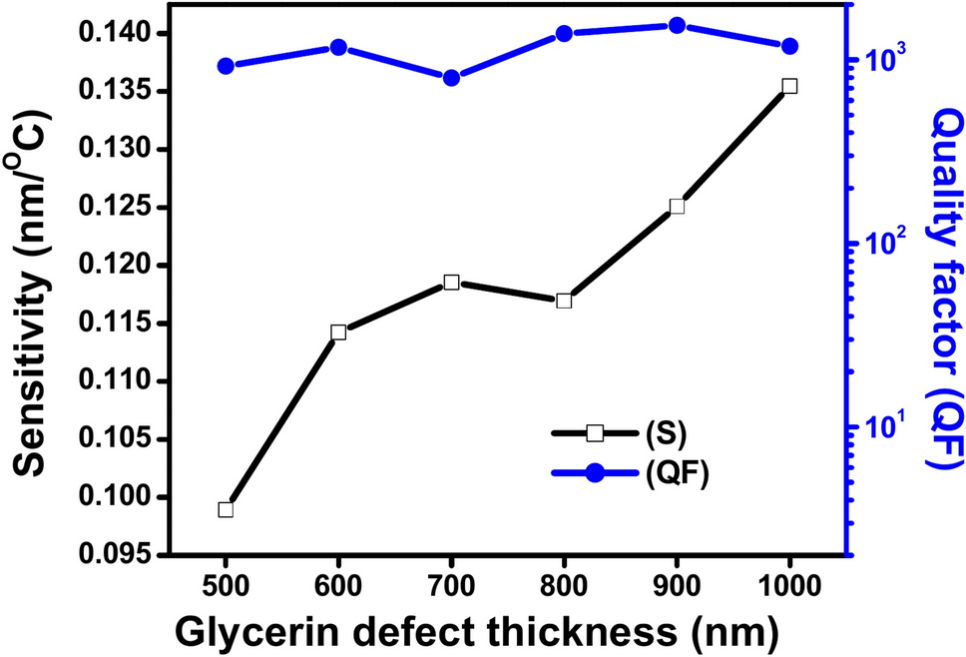
Shows the effect of change the glycerin central defect thicknesses D_def_, the sensitivity (black line) and the QF (blue line) at θ = 0º.

Figure 5 depicts the relationship between the glycerin central defect thickness D_def_, and both the sensitivity (in the black line) and the quality factor (QF, in the blue line) of the proposed 1D photonic crystal (PhC) thermal sensor at an incident angle of θ = 0°. The figure shows that the sensitivity generally increases as the defect thickness *D*def increases from 500 to 1000 nm, with some fluctuations. The sensitivity shows a gradual rise, reaching a peak around D_def_ of 800 nm before experiencing a slight decline at higher thicknesses. The increase in sensitivity with an increase in defect thickness is likely due to the enhanced interaction between the photonic crystal structure and the temperature changes as the defect layer becomes thicker causing an increment of the optical path of the defect layer, which leads to a more pronounced shift in the transmission spectrum. At the same time, the QF remains relatively stable as D_def_ increases, with minor fluctuations. It shows a slight increase from 500 to 800 nm, indicating improved resonance sharpness and fewer losses. After reaching its peak around D_def_ of 800–900 nm, the QF begins to decline slightly at the highest thickness (1000 nm). The overall behavior of the QF suggests that while increasing the defect thickness can improve the resonance quality up to a point, further increases may lead to a reduction in performance, possibly due to excessive absorption or scattering within the thicker defect layer. Finally, the figure indicates an optimal range for the defect thickness D_def,_ around 800–900 nm, where both sensitivity and QF are maximized. Beyond this range, further increases in thickness may diminish the quality factor while only marginally improving sensitivity.

### Effect of incident angle (θ)

**Fig. 6:**
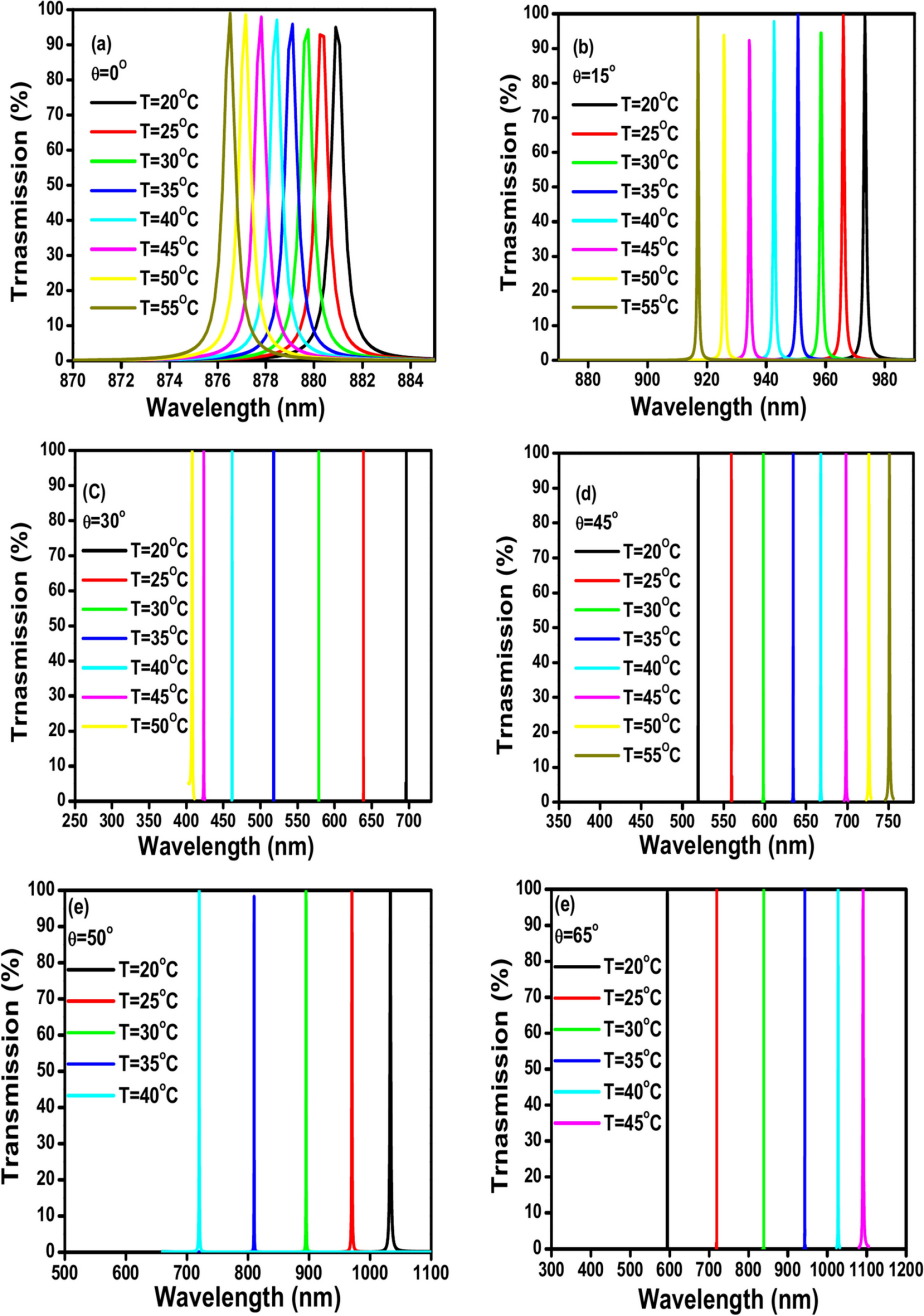
(**a**–**f**) Shows the TE transmission spectra of the proposed 1D PC thermal sensor at incident angles θ from 0 to 65º, With parameters: defect thickness D_def_ = 900 nm, GaN thickness d_A_ = 100 nm, air thickness d_B_ = 200nm and number of GaN/air periods repetitions N = 3; (**a**) θ = 0º, (**b**) θ = 15º, (**c**) θ = 30º, (**d**) θ = 45º, (**e**) θ = 50º, and (**f**) θ = 65º.

The proposed 1D PhC thermal sensor’s TE transmission spectra are displayed in Fig. 6a–f of the incidence angles of 0°, 15°, 30°, 45°, 50°, and 65°. The calculations are achieved for the defect thickness D_def_ of 900 nm, GaN thickness d_A_ of 100 nm, air thickness d_B_ of 200 nm, and the number of GaN/air period repetitions N = 3. Notably, the angle of incidence θ could not increase further to 65º due to practical considerations. Since the maximum incident angle in a PhC thermal sensor is limited by practical considerations, such as the performance of the sensor^26,47,48^. It has been established that the change in incidence angle, which produces sharper peaks at different temperatures, is the basic cause of the increase in sensitivity. Furthermore, there is a large shift between the peaks, which suggests an increase in the quality factor. Some of the TE transmission spectra show a red shift, while others show a blue shift. Red-shifted TE transmission spectra are observed at the incidence angles of 45º and 65º. Although the blue-shifted TE transmission spectra are observed at the incidence angles of 0º, 15º, 30º, and 50º. Notably, all the transmission spectra exhibit a red or blue shift in the visible and near-infrared spectrums.

Table 2 shows the sensing parameters sensitivity (S), quality factor (QF), figure of merit (FOM), signal-to-noise ratio (SNR), detection limit (δn), and sensor resolution (SR) for the proposed thermal sensor by increasing the incident angle θ from 0º to 65º. The data is calculated for the defect thickness D_def_ of 900 nm, the GaN thickness d_A_ = 100 nm, the air thickness d_B_ of 200 nm, and the number of GaN/air period repetitions N = 3. Also, the temperature range, wavelength range, and shift direction are detected for each angle. The data in the table suggests that there is a fluctuation between the calculated sensing parameters and an increase in the incident angle θ for the suggested 1D PhC thermal sensor. This is consistent with the general understanding that the performance of PhC sensors can be affected by the angle of incidence^26,47,48^. There isn’t a clear relationship between the incident angle (θ) and the calculated sensing parameters across the data points provided. However, there is no consistent increase or decrease in the sensing parameters as the incident angle increases. Notably, the sensing parameters are not allowed (NA) and cannot be calculated at some incident angles, such as θ = 10°, 40°, 55°, and 60°. These missing data points make it difficult to understand how the calculated sensing parameters change across the entire range of incident angles. For example, the sensitivity (S) increases when the incidence angle θ increases from 0° (0.125 nm/ºC) to 5° (0.297 nm/ºC). After that, it decreases from 15° (1.606 nm/ºC) to 20° (0.108 nm/ºC).

**Table 2:** Shows the values of the parameters sensitivity (S), quality factor (QF), figure of merit (FOM), signal-to-noise ratio (SNR), detection limit (δn), and sensor resolution (SR) at incident angles (θ) varies from 0 to 65º, defect thickness D_def_ = 900 nm, GaN thickness d_A_ = 100 nm, air thickness d_B_ = 200nm and number of GaN/air periods N = 3, with identification the temperature range, wavelength range and shift direction of each angle.

θ	S (nm/ºC)	QF	FOM (ºC^−1^)	SNR	δn (ºC)	SR (nm)	Temp. range	Wavelength range (nm)	Shift direction
0	0.125	1539.678	0.219	453.342	1016.438	120.498	20–55	873–884	Blue
5	0.296	1252.071	0.664	92.508	406.147	48.148	20–55	552–567	Red
10	NA	NA	NA	NA	NA	NA	NA	NA	NA
15	1.606	1427.766	2.436	118.820	119.618	14.180	20–55	910–985	Blue
20	0.108	13,816.943	2.333	455.307	1435.123	170.133	20–55	640–645	Red
25	2.308	254.881	1.234	240.089	44.811	5.312	20–55	420–530	Red
30	10.088	35,443.258	621.462	12,030.150	5.691	0.674	20–50	400–700	Blue
35	2.497	1550.879	5.196	1691.867	35.854	89.534	20–55	690–790	Blue
40	NA	NA	NA	NA	NA	NA	NA	NA	NA
45	6.648	4464.783	50.208	4024.852	15.004	37.468	20–55	520–760	Red
50	15.725	1535.228	28.376	333.752	9.601	150.986	20–40	700–1050	Blue
55	NA	NA	NA	NA	NA	NA	NA	NA	NA
60	NA	NA	NA	NA	NA	NA	NA	NA	NA
65	20.074	14,723.294	397.182	2174.234	6.821	136.946	20–45	593–1110	Red

Then it significantly increases from 25° (2.309 nm/ºC) to 30° (10.088 nm/ºC), which is the second highest value of sensitivity in the table. After that, it was at 35° (2.497 nm/ºC). Finally, it increases from 45° (6.648 nm/ºC) to 65° (20.075 nm/°C) which is the highest value of the sensitivity in the table. The quality factor (QF) also fluctuates in an inverse manner across the entire range of incident angles, comparing the sensitivity. The QF decreases from the angle 0° (1539.678) to the angle 5° (1252.071). Then it decreases from the angle of 15° (1427.766) to 20° (13,816.943). Then it significantly increases from the angle 25º (254.881) to 30º (35,443.258), which is the highest value of the quality factor in the table. After that, it dramatically decreases at the angle 35º (1550.879). Then it decreases again from the angle 45° (4464.783) to the angle 50° (1535.228), and finally it significantly increases at 65° (14,723.294), which is the second highest value of the quality factor in the table. With respect to the relationship between the increase in incident angles and the signal-to-noise ratio (SNR), It fluctuates significantly between different angles. It decreases from 453.342 at the angle 0° to 92.508 at the angle 5°. Then, there’s an increase at angle 15° (118.820) and again at angle 20° (455.307). After that, it decreases again at angle 25° (4024.852), but then it shows a significant increase at angle 30° (12,030.150). The trend continues with an increase in the incident angle from 35° (1691.867) to 45° (4024.852), then it shows a dramatic decrease at angle 50° (333.752), and finally it shows another increase at angle 65° (2174.234). Also, the detection limit (δn) shows a fluctuation with an increase in the incident angle as follows: It has a significant decrease in the detection limit from (1016.438 ºC) at the angle 0° to (119.618 ºC) at the angle 15°. Then it significantly increases at angle 20° (1435.123 ºC), which follows by a dramatic decrease at angle 30° (5.691 ºC). Generally, a lower detection limit (δn) is desirable for a sensor as it indicates higher sensitivity. Then it increases at angle 35° (35.854 ºC), but then decreases again at higher angles (45°, 50°, and 65°). Unfortunately, the missing data points at the angles 10°, 40°, 55°, and 60° make it difficult to say for certain about the trend at those intermediate angles. Noteworthy, the sensor resolution (SR) of the proposed 1D PhC thermal sensor has the same behavior as the detection limit (δn). However, SR dramatically decreases from (120.498 nm) at 0º to (14.180 nm) at the angle 15º. Then, it significantly increases at 20° (170.133 nm), which follows by dramatically decreasing to 30° (0.674 nm). Again, it significantly increases at the angle 35° (89.534 nm), which is followed by a decrease at 45° (37.468 nm). Finally, SR increases at 50° (150.986 nm), which follows another decrease at 65° (136.946 nm). The temperature range for the TE transmission spectra ranges from 20º to 55º. A red shift is observed in some TE transmission spectra, while a blue shift is observed in others. red-shifted TE transmission is detected at incident angles of 5º, 20º, 25º, 45º, and 65º. Despite this, the incidence angles of 0º, 15º, 30º, 35º, and 50º of the blue-shift TE transmission spectra are detected. It is important to note that the TE transmission spectra exhibit a red and blue shift in the visible light and near-infrared spectrums from 420 to 1110 nm. Interestingly, electromagnetic radiation possesses a low wavelength with a red shift and a high wavelength with a blue shift. Thus, based on the interpretation of the effect of the change in the incident angle (θ) in the sensing parameters of the suggested thermal sensor, θ = 65º is an optimal angle to attain the maximum sensitivity and quality factor of this specific sensor design.

### Effect of change of GaN thickness (d_A_)

#### At incident angle 30 º

Table 3 displays the calculations for sensitivity (S), quality factor (QF), figure of merit (FOM), signal-to-noise ratio (SNR), detection limit (δn), and sensor resolution (SR) at different GaN d_A_ thicknesses ranging from 100 to 900 nm. The data is calculated for defect thickness D_def_ of 900 nm, air thickness d_B_ of 200 nm, an incident angle of 30º and the number of GaN/air period repetitions N = 3. For each thickness of GaN, the temperature range, wavelength range, and shift direction are determined. Based on the data in Table 3, there is an inverse relationship between the increase in GaN thickness (d_A_) and the sensitivity (S) of the proposed 1D PhC thermal sensor. As GaN thickness (d_A_) increases from 100 to 900 nm, the sensitivity (S) generally decreases. The decreasing relationship is not perfectly linear. While the other calculated sensing parameters appear fluctuation whether increasing or decreasing across the different GaN thicknesses (d_A_) without a clear trend. For instance, the quality factor (QF) varies significantly across different GaN thicknesses. QF increases from 35,443.258 at 100 nm to 96,101.415 at 200 nm, then it drops significantly to 18,100.882 at 300 nm. Then, it increases again to 72,411.983 at 500 nm which follows by decrease to 42,374.683 at 600 nm. Then, it increases again to reach 76,593.859 at 800 nm and finally it decreases to 59,342.563 at 1000 nm. In the same manner, FOM fluctuates throughout the range of GaN thicknesses. It increases from 621.462 ºC^−1^ at 100 nm to 1590.882 ºC^−1^ at 200 nm, then dramatically drops to 177.159 ºC^−1^ at 300 nm. Then it increases again to attain 656.275 ºC^−1^ at 400 nm, which follows a decrease to 306.792 ºC^−1^ at 600 nm. Then, it increases again to 465.465 ºC^−1^ at 800 nm, and finally, it decreases to 334.081 ºC^−1^. Also, SNR shows a significant fluctuation between the increasing and decreasing across the increase GaN thickness. It significantly decreases from 12,030.150 at 100 nm to 2212.286 at 600 nm. Then, it increases to attain the highest value of SNR of 134,814.539 at 700 nm which dramatically decreases to 1240.809 at 800 nm which follows by slight increase to 1386.438 at 1000 nm. In the same way, the detection limit (δn) has the same behavior. It rises from 5.691 ºC at 100 nm to 13.242 ºC at 300 nm, then drops to 11.875 ºC at 400 nm. After that, it increases again to 24.954 ºC at 800 nm, which is the highest value of (δn), then slightly decreases to 22.617 ºC at 1000 nm. Also, sensor resolution (SR) significantly increases from 0.674 nm at 100 nm to 90.022 nm at 300 nm, then decreases to 70.080 nm at 400 nm. After that, it increases to 86.220 nm at 800 nm, and finally, it slightly decreases to 81.223 nm at 1000 nm. Notably, a higher signal-to-noise ratio (SNR) could result from a higher quality factor (QF). Likewise, a smaller detection limit may result in a higher sensitivity (S). Noteworthy, all TE transmission spectra occurred in the temperature range of 20º to 50º. Furthermore, all the resonances have a blue shift direction in the visible light region of 400 to 725 nm. Thus, based on the interpretation of the effect of the change in the GaN thickness on the sensing parameters of the suggested thermal sensor, (d_A_)100 is an optimal GaN thickness for maximizing the sensitivity (10.088 nm/ºC) of this specific sensor design.

**Table 3:** Shows the values of the parameters sensitivity (S), quality factor (Q), figure of merit (FOM), signal-to- noise ratio (SNR), detection limit (δn), and sensor resolution (SR) at different thickness of GaN d_A_ from100 to 900 nm, the air thickness d_B_ = 200 nm, the incident angle θ = 30º, number of GaN/air periods N = 3 and the defect thickness D_def_ = 900 nm with identification the temperature range, wavelength range and shift direction for each thickness.

d_A_	S (nm/ºC)	QF	FOM (ºC^−1^)	SNR	δn (ºC)	SR (nm)	Temp. range	Wavelength range (nm)	Shift direction
100	10.088	35,443.258	621.462	12,030.150	5.691	0.674	20–50	400–700	blue
200	8.158	96,101.415	1590.882	6675.526	7.275	59.357	20–45	410–610	blue
300	6.798	18,100.882	177.159	2528.812	13.242	90.022	20–40	610–725	blue
400	5.901	45,793.603	447.025	29,468.163	11.875	70.080	20–40	540–657	blue
500	5.008	72,411.983	656.275	2535.399	14.951	74.889	20–40	508–605	blue
600	4.747	42,374.683	306.792	2212.286	17.0004	80.708	20–35	610–682	blue
700	3.854	53,812.996	343.475	134,814.539	20.958	80.786	20–40	560–640	blue
800	3.455	76,593.859	465.465	1240.809	24.954	86.220	20–40	535–605	blue
900	3.591	59,342.563	334.081	1386.438	22.617	81.223	20–35	605–660	blue

**Fig. 7:**
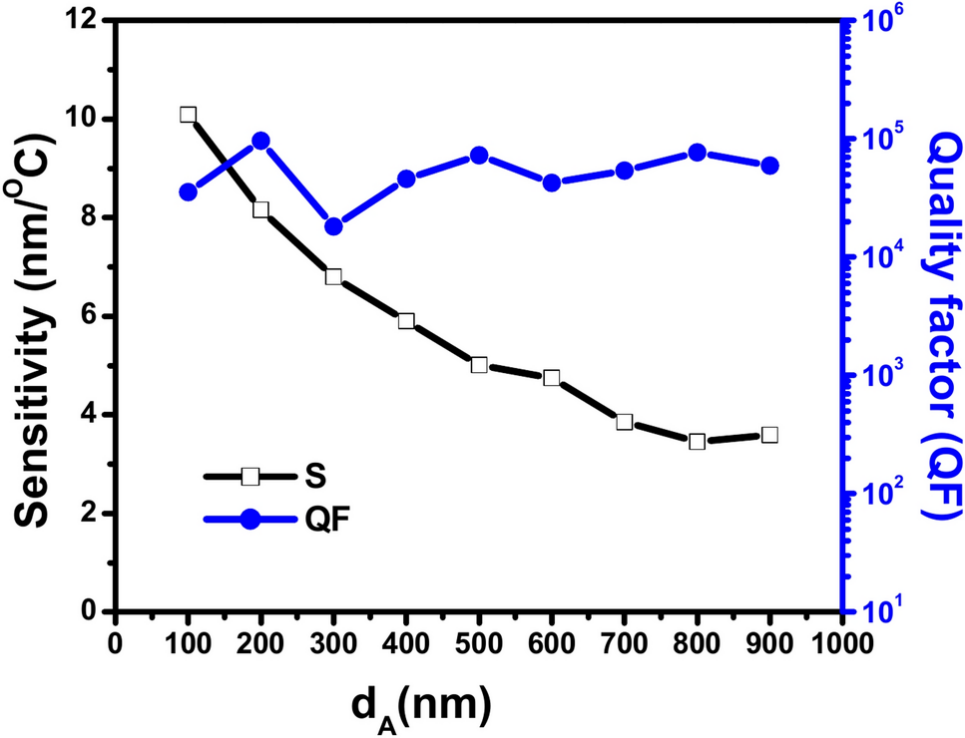
Shows the effect of the thickness of GaN (d_A_) on the sensitivity (black line) and the QF (blue line) at θ = 30º.

Figure 7 displays the relation between the sensitivity and the quality factor with increase the GaN thickness at incident angle of 30º. The figure shows beyond GaN thickness (d_A_) 100 nm the sensitivity begins to decline steadily, while the quality factor fluctuates but remains relatively high. Also, the figure shows the inverse relationship between the sensitivity and the GaN thickness. Whereas the sensitivity decreases with increased GaN thickness this is due to the multireflection processes between the whole layers of photonic crystal increases. Thus, the rate of the destructive interference process inside the photonic crystal increases. This leads to a reduction of sensitivity. At the same time, the quality factor increases initially, then it fluctuates with several peaks and troughs, and remains relatively high despite the increase in defect thickness. This indicates that while increasing GaN thickness reduces sensitivity, it helps in maintaining or improving the quality factor up to a certain extent.

#### At incident angle 65º

Table 4 displays the calculations for sensitivity (S), quality factor (QF), figure of merit (FOM), signal-to-noise ratio (SNR), detection limit (δn), and sensor resolution (SR) at different GaN d_A_ thicknesses ranging from 100 to 900 nm. The data is calculated for defect thickness D_def_ 900 nm, air thickness d_B_ 200 nm, an incident angle of 65º and the number of GaN/air period repetitions N = 3. For each thickness of GaN, the temperature range, wavelength range, and shift direction are determined. Table 4 shows that the sensitivity (S) of the suggested 1D PhC thermal sensor seems to be inversely correlated with the increase in GaN thickness (d_A_). Generally speaking, when GaN thickness (d_A_) increases from 100 to 900 nm, the sensitivity (S) drops; however, this decreasing relationship is not perfectly linear. Since the sensitivity increases slightly between 400 nm (10.827 nm/ºC) and 500 nm (11.186 nm/ºC). The data together imply that a thicker GaN layer may not always enhance the sensitivity of the sensor. This data set suggests that a GaN layer that is thinner—roughly 100 nm—might be better. While the relationship between the quality factor (QF) and the increase in GaN thickness (d_A_) of the proposed 1D PhC thermal sensor is difficult to describe. Since there isn’t simply an increase or decrease trend, and there is a variance in the quality factor (QF) with varying GaN thicknesses d_A_. Table 4 shows results showing a large variation in the quality factor (QF) for different GaN thicknesses. For example, it decreases to 2295.369 at 200 nm from 14,723.296 at 100 nm, followed by a rise sharply to 13,568.691 at 300 nm. At 500 nm, it again falls sharply to 3028.869 and then rises to 8914.900 at 600 nm. Eventually, it drops to 3725.620 at 800 nm, and then rises dramatically to 7029.010 at 900 nm. Also, the relationship between the increase in GaN thickness (d_A_) and the figure of merit (FOM) of the proposed 1D PhC thermal sensor appears complex. FOM also fluctuates throughout the increase in GaN thickness. It dramatically decreases from 397.187 ºC^−1^ at 100 nm to 32.355 ºC^−1^ at 200 nm, then significantly increases to 224.994 at 300 nm. It continues to fluctuate across the different GaN thicknesses. Since it significantly decreases again at 500 nm to 25.210 ºC^−1^, which follows by increasing another time to 83.504 ºC^−1^ at 600 nm, Finally, it decreases again to 22.146 ºC^−1^ at 800 nm, followed by another increase to 42.701 at 900 nm. SNR also fluctuates between increasing and decreasing as GaN thickness increases. It significantly drops from 2174.223 at 100 nm to 583.197 at 200 nm, followed by an increase to 1197.744 at 300 nm. Then, it decreases to 593.212 at 400 nm, followed by increasing to 680.414 at 500 nm. Then, it decreases again to 478.833 at 600 nm, followed by increasing to 1379.108 at 700 nm. Subsequently, it experienced another decrease, reaching 291.838 at 800 nm. Finally, it attained the highest value of 2308.629 at 900 nm. This suggests that a thicker GaN layer generally leads to a higher SNR in this sensor design. Similarly, there is not an apparent trend in the values of δn, which sometimes rises or decreases as the GaN thickness increases. It is 6.821 ºC at thickness 100 nm, while it is 11.260 ºC at thickness 200 nm. Then, it drops to 9.670 ºC at 300 nm, while it rises sharply to 21.063 ºC at 400 nm. Then, at 600 nm, it drops to 17.814 ºC, and at 800 nm, it dramatically increases to 30.496 ºC. At 900 nm, it finally drops significantly to 30.181 ºC nm. Furthermore, the relationship between SR and the increase in GaN thickness does not show a simple increase or decrease pattern. Since the sensor’s resolution values fluctuate as GaN thickness increases. According to the data in Table 4, SR value increases from 136.945 nm to 203.271 nm when GaN thickness increases from 100 to 200 nm. Then it decreases to 136.016 nm at 300 nm, followed by an increase again to 228.078 nm at 400 nm. After that, SR’s value decreases dramatically to attain the lowest value of SR in the table 2.111 nm at 600 nm. Then, it was followed by a large increase to 245.470 nm at 800 nm, which is the highest value of SR, followed by a significant decrease to 205.982 nm at 900 nm. It is important to note that all TE transmission spectra occurred in the temperature range of 20º to 50º and exhibit a red shift in the visible light and near-infrared spectrums from 593 to 1063 nm.

**Table 4:** Shows the values of the parameters sensitivity (S), quality factor (QF), figure of merit (FOM), signal-to-noise ratio (SNR), detection limit (δn), and sensor resolution (SR) at different thickness of GaN (d_A_) from100 to 900 nm, the air thickness d_B_ = 200 nm, the incident angle θ = 65º, number of GaN/air periods N = 3 and the defect thickness D_def_ = 900 nm with identification the temperature range, wavelength range and shift direction for each thickness.

dA	S (nm/ºC)	QF	FOM (ºC^−1^)	SNR	δn (ºC)	SR (nm)	Temp. range	Wavelength range (nm)	Shift direction
100	20.074	14,723.296	397.187	2174.223	6.821	136.945	20–45	593–1105	Red
200	18.051	2295.369	32.355	583.197	11.260	203.271	30–55	1045–1508	Red
300	14.065	13,568.691	224.994	1197.744	9.670	136.016	25–40	771–986	Red
400	10.827	4600.163	45.061	593.212	21.063	228.078	30–55	955–1280	Red
500	11.186	3028.869	25.210	680.414	18.447	206.352	35–55	1225–1456	Red
600	9.706	8914.900	83.504	478.833	17.814	2.111	30–45	973–1122	Red
700	7.769	5066.221	32.704	1379.108	26.902	209.016	35–55	1140–1305	Red
800	8.049	3725.621	22.146	291.838	30.496	245.470	35–55	1262–1432	Red
900	6.824	7029.010	42.701	2308.629	30.181	205.982	30–50	1057–1063	Red

**Fig. 8:**
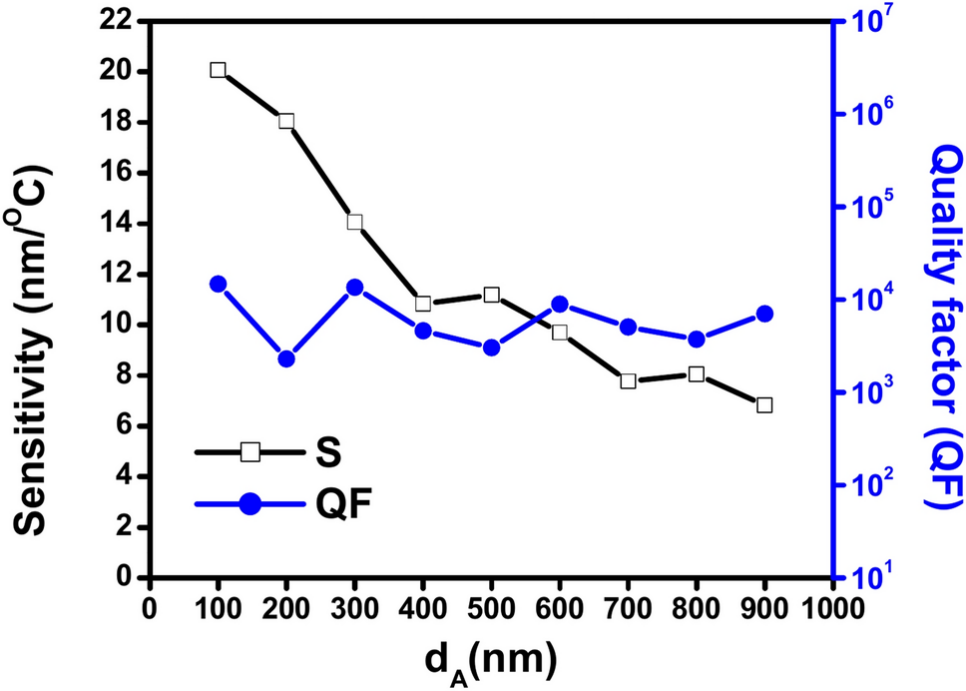
Shows the effect of the thickness of GaN (d_A_) on the sensitivity (black line) and the QF (blue line) at θ = 65º.

Figure 8 presents the relation between the sensitivity and the quality factor with an increase in the GaN thickness at incident angle of 65º. The figure shows the sensitivity decreases with increase the GaN thickness. This is also due to the multireflection processes between the whole layers of photonic crystal increases which leads to a reduction of the sensitivity value. However, the rate of the destructive interference processes inside the photonic crystal at incident angle 65º increases than the incident angle 30º. At the same time, the quality factor is almost constant with increasing the GaN thickness as the same in the incident angle 30º.

### Effect of change number GaN/air periods repetitions (N)

#### At incident angle 30º

**Fig. 9:**
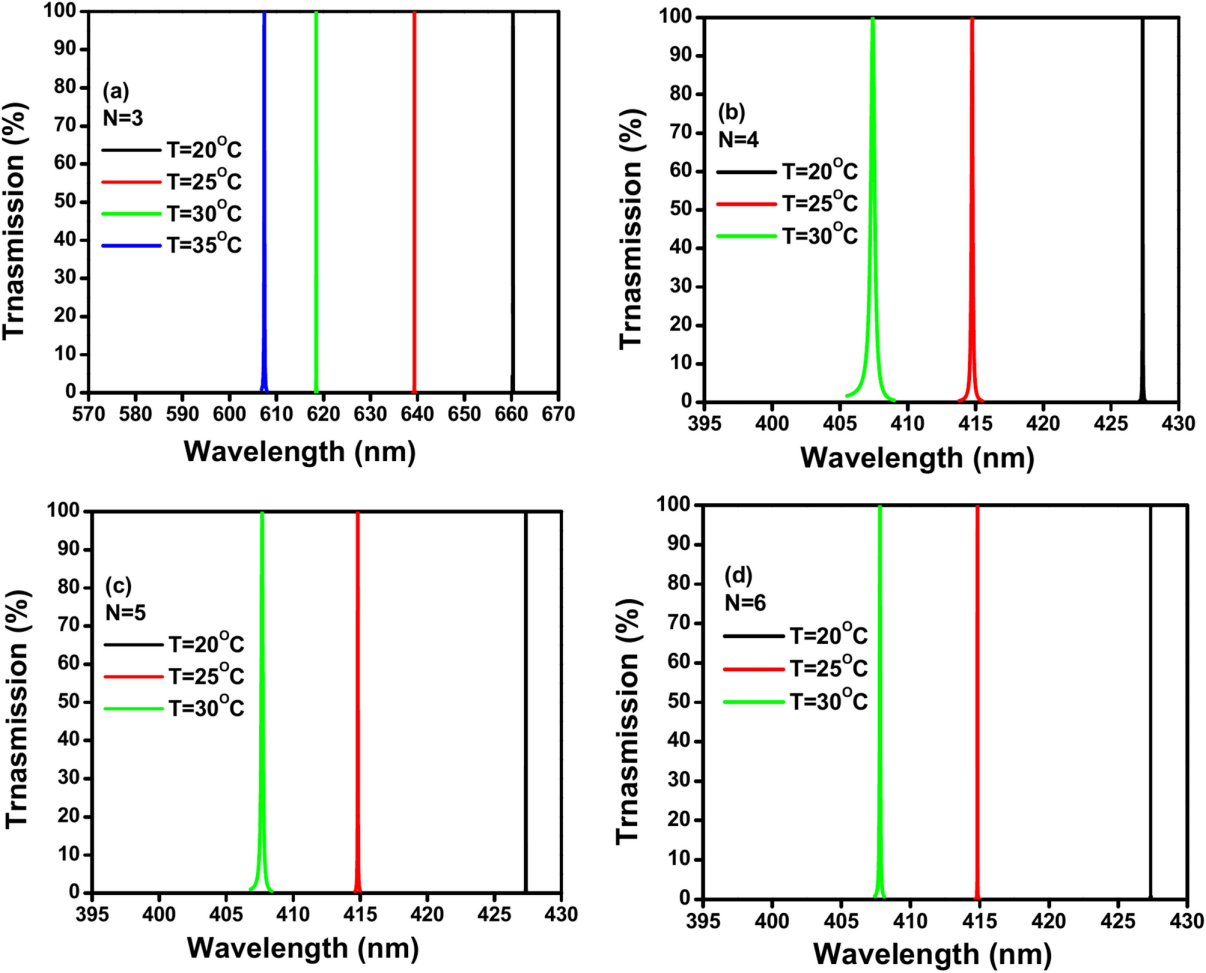
(**a**–**d**) Shows the TE transmission spectra of the proposed 1D PC thermal sensor at different number of GaN/air periods, incident angles θ = 30º, defect thickness D_def_ = 900 nm, GaN thickness d_A_ = 100 nm, air thickness d_B_ = 200nm: (**a**) N = 3, (**b**) N = 4, (**c**) N = 5, (**d**) N = 6.

Figure 9a–d shows TE transmission spectra of the proposed 1D PhC thermal sensor at different temperatures, with the following parameters: incident angle θ = 30°, defect thickness D_def_ = 900 nm, GaN thickness d_A_ = 100 nm, air thickness d_B_ = 200 nm, and different GaN/air period repetitions (N) from 3 to 6. The different lines on the graph represent the transmission spectra at different temperatures: black line: 20 °C, red line: 25 °C, green line: 30°C and blue line: 35 °C. Each colored line (black, red, green, blue) exhibits a peak at a specific wavelength, indicating the resonant wavelength where maximum transmission occurs. The figure shows as the temperature increases the TE transmission peaks shift towards shorter wavelengths (blue shift). This behavior indicates a direct relationship between temperature and the position of the transmission peak: as the temperature rises, the resonant wavelength decreases. The shift in transmission peaks with temperature is likely due to the temperature dependence of the refractive index of the materials (GaN and air) used in the photonic crystal structure, causing changes in the photonic band gap. The figure also shows the TE transmission spectra shows a blue shift as the number of GaN/air period repetitions (N) increases from 3 to 6. With fewer period repetitions (N = 3), the resonance peaks are well-separated, indicating higher sensitivity to temperature changes. As the number of period repetitions increases (N = 4, N = 5, N = 6), the peaks move closer together, indicating reduced sensitivity to temperature changes. The wavelength range shifts from a broader range (570–670 nm for N = 3) to a narrower range (395–430 nm for N = 4, N = 5, and N = 6). The separation between the peaks at different temperatures decreases with increasing N, suggesting that a lower number of period repetitions might be more advantageous for achieving higher sensitivity in this thermal sensor design.

Table 5 shows the sensing parameters that are calculated of the proposed 1D PhC thermal sensor for different numbers of GaN/air period repetitions N = 3, 4, 5 and 6 at incident angle 30º. The table contains different values of sensing parameters of the proposed sensor: sensitivity (S), quality factor (QF), figure of merit (FOM), signal-to-noise ratio (SNR), detection limit (δn) and sensor resolution (SR). Also, the table shows the temperature range, wavelength range, and shift direction for each number of GaN/air period repetitions. According to the data in the table, the number of period repetitions (N) is inversely correlated with the sensitivity (S). Whereas by increasing the number of GaN/air period repetitions (N) from 3 to 6 the sensitivity (S) decreases significantly. Since the most considerable drop occurs between N = 3 and N = 4, with sensitivity dropping from 10.088 nm/°C to 2.004 nm/°C. while from N = 4 to N = 6, the sensitivity continues to decrease but at a much smaller rate. This suggests that increasing the number of GaN/air period repetitions reduces the sensor’s sensitivity to temperature changes, with the most significant reduction happening between number of period repetitions N = 3 and 4. While the data in the table indicates a non-linear relationship between the quality factor and the number of GaN/air period repetitions. Initially, the increase of the number of period repetitions from 3 to 4 reduces the value of the quality factor sharply from 35,443.258 to 7419.528, but further increases in the number of period repetitions from 4 to 6 result in a substantial improvement in the quality factor, with the highest value of the quality factor achieved at N = 6 which is 250,132.271. By the same way, the data in the table indicates a non-linear relationship between the figure of merit and the number of GaN/air period repetitions. Initially, increasing the number of period repetitions from 3 to 4 results in a significant drop in the value of FOM from 621.462 to 35.066 °C^−1^. However, further increasing the number of period repetitions from 4 to 6 results in a significant increase in the FOM, with the highest FOM observed at N = 6 which is 1148.198 °C^−1^. Also, the data provided in the table indicates a non-linear relationship between the signal-to-noise ratio and the number of GaN/air period repetitions. Initially, increasing the number of period repetitions from 3 to 4 results in a substantial decrease in the SNR value from 12,030.150 to 93.567. However, further increasing the number of period repetitions from 4 to 6 leads to a significant increase in the SNR value. Also, the data provided in the table indicates a non-linear relationship between the number of GaN/air period repetitions and the detection limit. Initially, increasing the number of period repetitions from 3 to 4 results in a substantial increase in the detection limit value from 5.691 to 47.588 °C indicating to reduced sensitivity. However, further increasing the number of period repetitions from 4 to 6 leads to a decrease in the detection limit, though it does not reach the low level observed at N = 3 which indicates to increase sensitivity at this number of GaN/air period repetitions. Besides, the data in the table indicates a non-linear relationship between the sensor resolution and the number of GaN/air period repetitions. Initially, increasing the number of period repetitions from 3 to 4 results in a substantial increase in the sensor resolution from 0.674 to 95.367 nm. This sharp increase in the sensor resolution means that the sensor becomes less precise and indicates a decrease in resolution quality. However, further increasing the number of period repetitions from 4 to 6 leads to a decrease in the sensor resolution, thereby improving the precision, although it does not reach the high precision observed at N = 3. With respect to the operating temperature range of the proposed sensor. The data in the table indicates that as the number of GaN/air period repetitions increases from 3 to 4, the temperature range of the sensor narrows significantly. For N = 3, the sensor operates over a broader temperature range (20–50 °C). While beyond N = 4, increasing the number of period repetitions to 5 and 6 does not further affect the temperature range, which remains at temperature range narrows to 20–30 °C. Finally, the data in the table shows as the number of GaN/air period repetitions increases from 3 to 4, the wavelength range narrows significantly from a broad range (400–700 nm) to a much narrower range (405–428 nm). This range is included in the visible light spectrum (approximately 380–750 nm). While further increasing the number of period repetitions to 5 and 6 results in a slight additional narrowing of the wavelength range (405–427) which are within the blue to green region of the visible spectrum. Therefore, the sensor’s wavelength range covers the visible light spectrum, with narrower ranges focused more specifically within the blue to green light spectrum as the number of period repetitions increases and the shift direction remains consistently blue across all the different numbers of GaN/air period repetitions.

**Table 5:** Shows the values of the parameters sensitivity (S), quality factor (Q), figure of merit (FOM), signal-to-noise ratio (SNR), detection limit (δn), and sensor resolution (SR) at different number of periods from N = 4 to N = 6. At the condition GaN thickness d_A_ = 100 nm, air thickness d_B_ = 200 nm, incident angle θ = 30º, and defect thickness D_def_ = 900 nm with indentification the temperature range, wavelength range and shift direction for each thickness.

N	S (nm/ºC)	QF	FOM (ºC^−1^)	SNR	δn (ºC)	SR (nm)	Temp. range	Wavelength range (nm)	Shift direction
3	10.088	35,443.258	621.462	12,030.150	5.691	0.674	20–50	400–700	Blue
4	2.004	7419.528	35.066	93.567	47.588	95.367	20–30	405–428	Blue
5	1.967	42,018.859	194.318	288.659	38.770	76.265	20–30	405–427	Blue
6	1.956	250,132.271	1148.198	957.520	31.022	60.696	20–30	407- 427	Blue

**Fig. 10:**
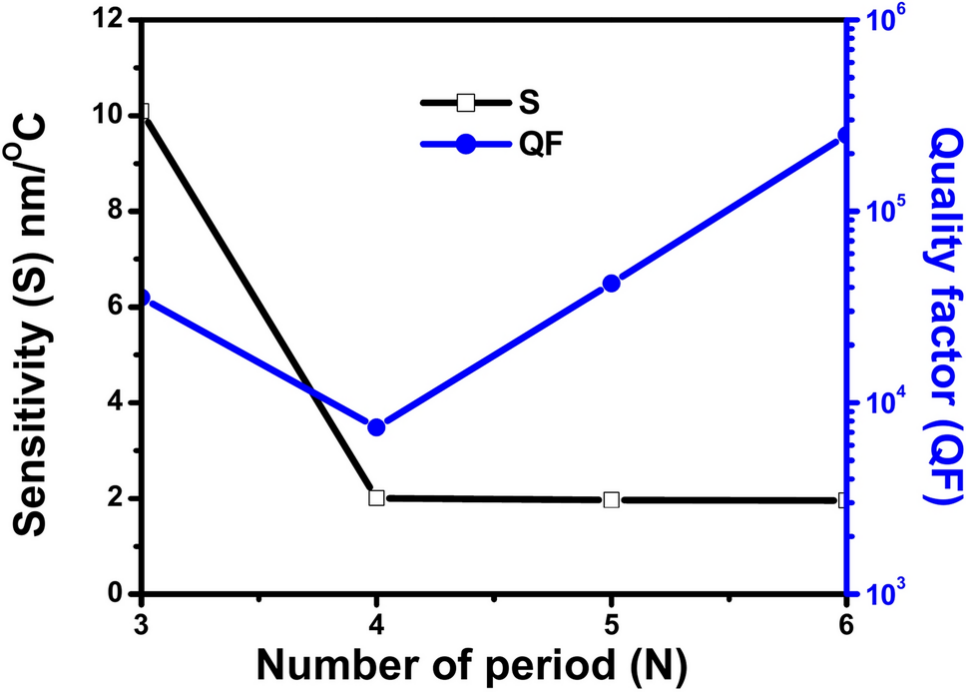
Shows the Effect of the number of (GaN/Air) periods (N), sensitivity (black line) and QF (blue line) at θ = 30º.

Figure 10 presents a line graph illustrating the relationship between sensitivity (S) and quality factor (QF) with respect to the number of GaN/air period repetitions at the incident angle 30°. The figure shows a significant decrease in the sensitivity value from 3 to 4 period repetitions, then it remains stabilizing at a low value from 4 to 6 period repetitions. While the figure shows a clear positive correlation between the number of period repetitions and the quality factor, since it shows a steady increase of the quality factor with increase of the number of period repetitions. Finally, the figure demonstrates a clear inverse relationship between the sensitivity and the number of GaN/air period repetitions, while the quality factor shows a direct correlation.

#### At incident angle 65º

**Fig. 11:**
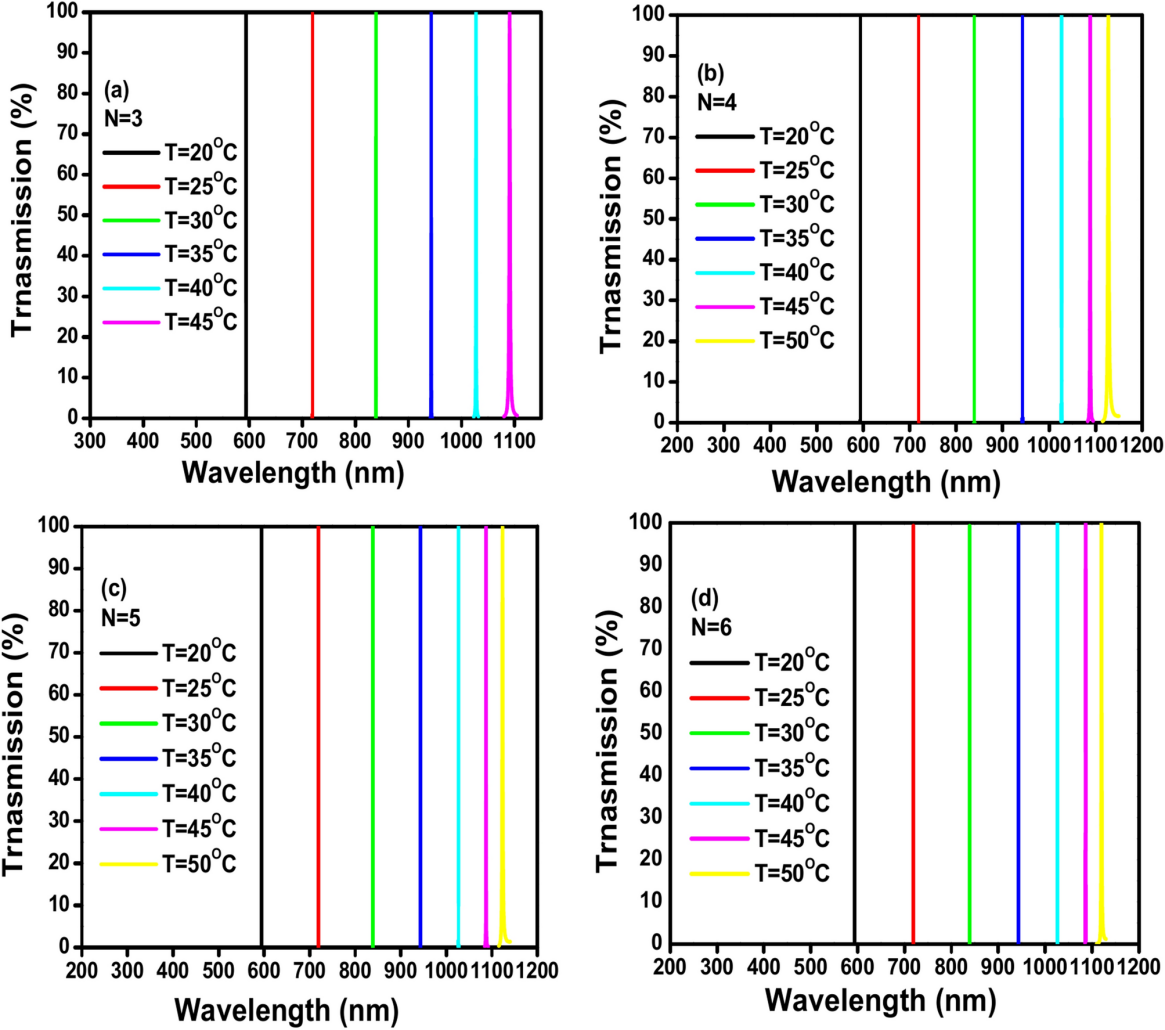
(**a**–**d**) Shows the TE transmission spectra of the proposed 1D PC thermal sensor at different number of GaN/air periods, incident angles θ = 65º, defect thickness D_def_ = 900 nm, GaN thickness d_A_ = 100 nm, air thickness (d_B_) 200nm: (**a**) N = 3, (**b**) N = 4, (**c**) N = 5, (**d**) N = 6.

The suggested 1D PhC thermal sensor’s TE transmission spectra are displayed in Fig. 11 (a-d) for different period repetitions ranging from 3 to 6 at angle θ = 65º, defect thickness D_def_ = 900 nm, GaN thickness d_A_ = 100 nm, and air thickness d_B_ = 200 nm. It is observed that the temperature range’s upper limit rises to 50ºC as the number of period repetitions increases from 3 to 6. Furthermore, the resonances of all N values exhibit a red shift direction in the visible and at the beginning of the near-infrared wavelength range (from 593 to 1110 nm). This implies that the peak of the response curve of the sensor might be shifting towards longer wavelengths.

Table 6 displays the values of different sensing parameters that are calculated of the proposed 1D PhC thermal sensor for different numbers of GaN/air period repetitions N = 3, 4, 5 and 6 at incident angle 65º. The table contains different values of sensing parameters as follows: sensitivity (S), quality factor (QF), figure of merit (FOM), signal-to-noise ratio (SNR), detection limit (δn), and sensor resolution (SR). Also, the table shows the temperature range, wavelength range, and shift direction for each number of period repetitions. Based on the data in the table, the number of period repetitions (N), sensitivity (S), and sensor resolution (SR) are inversely correlated. Based on the data in the table it is noticed that the number of period repetitions (N) increases from 3 to 6, the sensitivity (S) drops from 20.074 nm/°C to 17.868 nm/°C and the sensor resolution (SR) drops from 136.946 to 97.696 nm. Although the quality factor (QF) and the figure of merit (FOM) are directly correlated with the number of period repetitions (N). The quality factor (QF) increases dramatically from 14,723.294 to 13,824,513.53 and FOM increases significantly from 397.18 °C^−1^ to 3.39 × 10^5^ °C^−1^ as the number of period repetitions (N) increases from 3 to 6. While the signal-to-noise ratio (SNR) fluctuates across increasing numbers of period repetitions (N). Notably, it seems that the detection limit (δn) has a complicated relationship with the increase of the number of period repetitions (N). The values of the detection limit (δn) are very close at N = 4 and 5 whereas the lower detection limit (δn) for N = 6 is 5.467 ºC. Lastly, its lowest detection limit (δn) of 5.467 ºC is reached at N = 6. Thus, based on the interpretation of the effect of the number of GaN/Air period repetitions in the sensing parameters of the suggested thermal sensor, d_A_ = 100 nm is an optimal GaN thickness to attain the maximum sensitivity of this specific sensor design.

**Table 6:** Shows the Values of the parameters sensitivity (S), quality factor (Q), figure of merit (FOM), signal-to-noise ratio (SNR), detection limit (δn), and sensor resolution (SR) at different number of GaN/air periods from N = 4 to N = 6, at the condition GaN thickness d_A_ = 100nm, air thickness d_B_ = 200 nm, incident angle θ = 65º, and defect thickness D_def_ = 900 nm with identification the temperature range, wavelength range and shift direction for each thickness.

N	S (nm/ºC)	QF	FOM (ºC^−1^)	SNR	δn (ºC)	SR (nm)	Temp. range	Wavelength range (nm)	Shift direction
3	20.074	14,723.294	397.182	2174.234	6.821	136.946	20–45	593–1110	Red
4	18.047	125,332.262	3073.591	13,103.113	7.798	140.745	20–50	594–1150	Red
5	17.936	1,016,709.768	2.58 × 10^4^	36,921.667	7.549	135.411	20–50	594–1150	Red
6	17.868	13,824,513.53	3.39 × 10^5^	788,425.878	5.467	97.696	20–50	594 -1150	Red

**Fig. 12:**
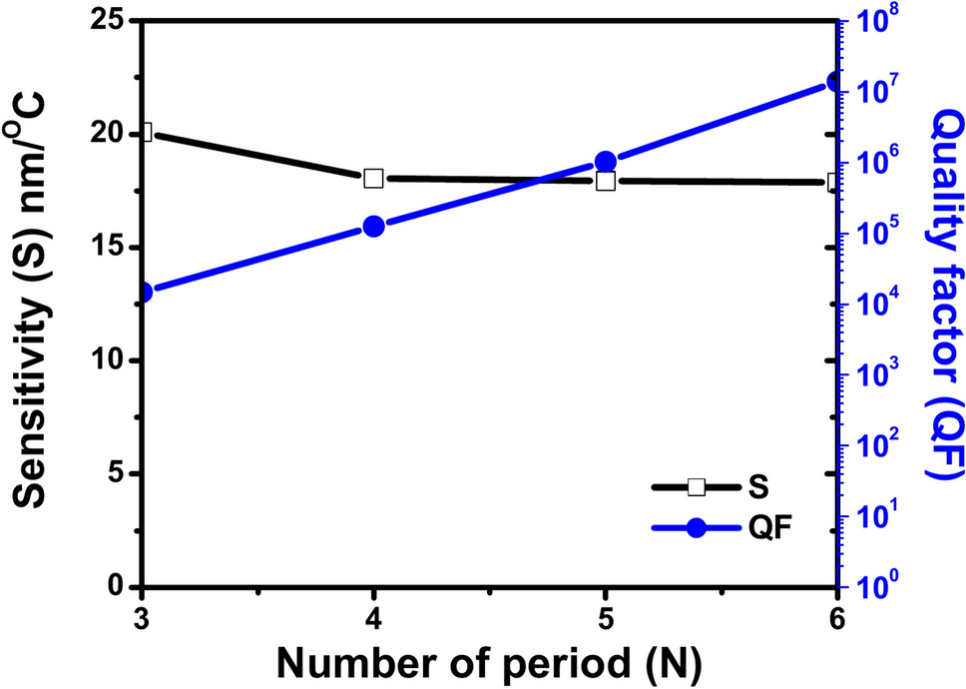
Shows the effect of change number (GaN/Air) periods (N), sensitivity (black line) and QF (blue line) at θ = 65º.

Figure 12, the black line illustrates how little the impact of the number of GaN/Air period repetitions on the suggested 1D PhC’s sensitivity. Since there is a small drop in sensitivity value from 20.074 nm/ºC for N = 3 to 17.868 nm/ºC for N = 6. However, the quality factor (blue line) shows a high increase from 14,723.294 for N = 3 to 13,824,513.53 for N = 6 with temperature range 20-50ºC.

## Experimental approach

In this study, the structure of the proposed 1D PhC thermal sensor is established using the GaN dielectric material. Moreover, Glycerin is introduced into the faulty area for thermal sensing purposes. Dry and wet etching methods can be used to fabricate this stracture^63–67^. The photolithography technique can be used to open up the grooved GaN structure^68^. The photolithography method is used to create 1D PhC systems by opening the air gaps between the GaN walls. T.S. Perova et.al^69^ applied the optofluidic microsystem (OFM) technique to infiltrate a NLC of type E7 into the air spaces between the Si walls^70^. Based on the research by S. Surdo et.al^70^, the proposed current 1D PhC thermal sensor system can infiltrate glycerin into the central defect region of the 1D PhC system using the OFM technique.

## Conclusion

In conclusion, the transfer matrix (TMM) approach was used to investigate the thermal characteristics of the suggested construction. The proposed structure is made up of a defective one-dimensional photonic crystal thermal sensor composed of gallium nitride (GaN), glycerin, and air layers. The effect of defect thickness Ddef, incident angle θ, the GaN thickness dA, and the number of GaN/air period repetitions (N) are investigated on the different sensing parameters of the proposed sensor. The study suggests that the optimal glycerin defect thickness for a thermal sensor is 900 nm, as it enhances sensitivity and quality factors. The proposed thermal sensor’s TE transmission spectra were analyzed at angles from 0° to 65°, with a maximum angle of incidence of 65° due to practical performance issues. Spectra shows redshifts at higher angles and blueshifts at lower angles. The sensitivity fluctuates with angle changes, peaking at 65° with the highest value recorded. The sensing parameters like sensitivity, quality factor, and signal-to-noise ratio fluctuate with angle changes, with the optimal angle being 65° for maximum sensitivity and quality factor. The study explores the impact of GaN thickness on thermal sensor performance at different incident angles. It reveals a relationship between temperature and resonant wavelength, and a decrease in sensitivity as GaN thickness increases. The optimal GaN thickness is 100 nm. The study also explores the impact of GaN/air period repetitions on a thermal sensor’s performance at the incident angles. As the temperature increases, transmission peaks shift to shorter wavelengths, indicating a blue shift. Fewer GaN/air period repetitions (N = 3) show better temperature sensitivity. Sensitivity decreases with an increase of GaN/air, the quality factor and figure of merit also improve. The operating temperature range narrows, focusing more on the blue-to-green light spectrum. The optimal number of GaN/air period repetitions is N = 3. The small number of period repetitions helps to create a compactable sensor. Our findings indicate that the suggested sensor structure has a sensitivity of 20 nm/°C, a high-quality factor of 14,723, the figure of merit of 397 ºC^−1^, a signal-to-noise ratio of 2174, a detection limit of 7 °C, and a sensor resolution of 137 nm. Finally, by comparison of the present work results with the previous thermal sensor studies as shown in Table 7, our findings represented the highest values of sensitivity, quality factor and figure of merit. Thus, the present work suggests a sensor that more helpful in the creation of cheap and practical thermal sensors.

**Table 7:** Shows the comparison between the performance of our sensor and some reported works.

Ref	Structure	S (nm/ºC)	QF	FOM (°C^−1^)	SNR	Δn (°C)	SR (nm)	Operating range
^13^	[(TiO_2_)^6^ SiO_2_ (SiO_2_)^6^]	0.0037	NA	NA	NA	NA	NA	0–300 ºC
^13^	[(TiO_2_)^6^ Bi_4_Ge_3_O_12_ (SiO_2_)^6^]	0.005	NA	NA	NA	NA	NA	0–300 ºC
^33^	[Si/air)^5^ Si (air/Si)^5^]	0.064	NA	NA	NA	NA	NA	100:700 k
^34^	Cascaded Si (PhC) nanobeam cavities	0.1629	4 × 10^4^	NA	NA	0.08	NA	30:80 °C
^15^	[(Si)(air/Si)^3^(LC)(Si/air)^3^(Si)]	0.328	4840	NA	NA	NA	NA	15:55 °C
^35^	[Si/polymer/SiO_2_]	0.380	NA	NA	NA	NA	NA	25:100 ºC
^36^	[(Al_2_O_3_)^5^ Toluene (TiO_2_)^5^]	0.0887	471.4	NA	NA	NA	NA	− 20:70 °C
^37^	[(TiO_2_/Al_2_O_3_)^N^/Al_2_O_3_/(TiO_2_/Al_2_O_3_)^N^]	0.0065	NA	NA	NA	NA	NA	0:300 ºC
^38^	[Si/SiO_2_)^10^ (MLC-9200-000) (Si/SiO_2_)^10^]	0.224	NA	20.36	814	0.006	0.0014	15:55 ºC
^39^	[Si/SiO_2_)^N/2^ TiO_2_ (Si/SiO_2_)^N/2^]	0.01069	NA	0.218	218.2	0.795	0.0085	0:1000 ºC
^40^	Air (SiO_2_/Si)^N1^(G/LC/G) (SiO_2_/Si)^N2^ substrate G/LC/G defect (LC thickness 2500 nm)	4	11,000	NA	NA	NA	NA	15:55 ºC
^37^	prism/Au/water/(Si∕SiO_2_)^N^/Si	2.8:10.8	3.5 × 10^3^	NA	NA	3 × 10^−7^	NA	0–100 ºC
^38^	(Si/SiO_2_/PS)^N^ with N = 10	0.08758	NA	NA	NA	NA	NA	25–100 ºC
^26^	Si/PS/SiO_2_	0.085	2216.6	0.0243	NA	NA	NA	25–900 ºC
^44^	[air/(AB)^N/2^ D (AB)^N/2/^air] (Sensor-1) [air/(AB)^N/2^ (BA)^N/2^/air] (Sensor-2) [air/(AB)^N/2^ D (BA)^N/2^/air] (Sensor-3)	0.173	2284	NA	NA	NA	NA	25–375 ºC
^45^	[(Si)^6^NLC(SiO_2_)^6^]	− 0.12317	10^7^	1002.48	NA	NA	NA	0–50 ºC
Presented work	(GaN/Air)^N^GaN/Gylecrin/GaN(Air/GaN)^N^	20.074	14,723.2	397.18	2174.2	6.8218	136.946	20–45 ºC

## Data Availability

The datasets used and/or analyzed during the current study are available from the corresponding author on reasonable request.
